# Adverse events following administration of COVID-19 vaccines in Saudi Arabia

**DOI:** 10.1038/s41598-022-23471-8

**Published:** 2022-11-15

**Authors:** Saleh Alqahtani, Hani Jokhdar, Jaffar A. Al-Tawfiq, Salah Al-Otaibi, Abdullah Assiri, Sami Almudarra, Khaled Alabdulkareem, Alhan Haji

**Affiliations:** 1 Department of Medicine, King Faisal Specialist Hospital & Research Center, Riyadh, Saudi Arabia; 2 Division of Gastroenterology and Hepatology, Johns Hopkins University, Baltimore, MD USA; 3 Deputyship of Public Health, Ministry of Health, Riyadh, Saudi Arabia; 4 Specialty Internal Medicine and Quality Patient Safety Department, Johns Hopkins Aramco Healthcare, Dhahran, Saudi Arabia; 5 Infectious Diseases Division, Department of Medicine, Indiana University School of Medicine, Indianapolis, IN USA; 6 Infectious Diseases Division, Department of Medicine, Johns Hopkins University School of Medicine, Baltimore, MD USA; 7 Department of Anesthesia, King Faisal Specialist Hospital & Research Center, Riyadh, Saudi Arabia; 8 Assisting Deputyship for Preventive Health, Ministry of Health, Riyadh, Saudi Arabia; 9 Assisting Deputyship for Primary Health Care, Ministry of Health, Riyadh, 11176 Saudi Arabia

**Keywords:** Diseases, Infectious diseases, Viral infection

## Abstract

Previous studies investigated the frequency of different adverse events of COVID-19 vaccines. However, this study compares these adverse events between the two main COVID-19 vaccines used in Saudi Arabia (Pfizer-BioNTech and Oxford-AstraZeneca) using telemedicine technology. A cross-sectional study was conducted among 958 individuals, 7 days after receiving either Pfizer-BioNTech or Oxford-AstraZeneca vaccines during June 2021. Immediate adverse events were reported by 1.04% and 2.09% for Pfizer-BioNTech and Oxford-AstraZeneca vaccines, respectively, with no serious events. Recipients of Pfizer-BioNTech vaccine had a higher percentage of local adverse events (24.8% versus 9.8% in AstraZeneca vaccine). The most common reported systemic adverse events in both vaccines respectively were general fatigue (23.1% and 25.1%), fever (18.5% and 27.2%), myalgia (20.6% and 20.3%), and headache (15.2% and 17.2%). No significant difference was recorded between both vaccines regarding overall systemic adverse events; however, they were more frequent following the first dose of AstraZeneca vaccine compared to Pfizer-BioNTech vaccine, while the reverse was observed for the second dose. Adverse events were more frequent in females and younger age groups for both vaccines. Most of systemic and local adverse events were mild in nature. Further cohort studies are recommended to investigate the long-term adverse events of COVID-19 vaccines.

## Introduction

Coronavirus disease 2019 (COVID-19) is an infectious disease caused by severe acute respiratory syndrome coronavirus 2 (SARS-CoV-2)^1^. As of Sept 24, 2021, there were about 412 million confirmed cases of COVID-19, and about 5.8 deaths, reported to World Health Organization (WHO) worldwide^2^, along with devastating medical, economic, and social consequences^3^.

Population-based vaccination is the best way to achieve herd immunity and prevent disease and community spread of infection^4^. Vaccines can reduce susceptibility among the uninfected and reduce the viral spread in those who are infected^5^.

The most commonly approved and used vaccines in the kingdom of Saudi Arabia are Pfizer-BioNTech and Oxford-AstraZeneca (AZD1222)^6^. Pfizer-BioNTech COVID-19 (BNT162b2) vaccine is a lipid nanoparticle-formulated, nucleoside-modified mRNA vaccine encoding the prefusion spike glycoprotein of SARS-CoV-2. Two doses of the vaccine produce high SARS-CoV-2 neutralizing antibody titers and robust antigen-specific CD8+ and Th1-type CD4+ T-cell responses^7^.

Oxford-AstraZeneca (AZD1222) vaccine is a replication-deficient chimpanzee viral vector based on a weakened version of a common cold virus (adenovirus) that causes infections in chimpanzees. It contains the genetic materials of the spike protein. After vaccination, the cells produce the spike protein, stimulating the immune system to attack the SARS-CoV-2 virus^8^.

On Aug 2021, 20,906, 174 (60.1%) of Saudi population were vaccinated with at least one dose of COVID-19 vaccine and 11,374,999 (32.0%) were fully vaccinated as documented by Saudi Ministry of Health (MOH)^9^.

The three most frequent recorded immediate adverse events (within the first fifteen minutes) for COVID-19 vaccines included vagal response (30%), anxiety reaction (24%) and dizziness (21%)^10^.

According to the Food and Drug Administration (FDA), the most common adverse events among participants in the Pfizer-BioNTech phase 3 clinical trials were short-term local responses including pain, redness and swelling at the injection site. Systemic responses included vomiting, diarrhea, fatigue, headache, muscle pain, chills, joint pain, and fever^8,11^. Reported serious adverse events included acute myocardial infarction and cerebrovascular lesions, as thrombosis. Unsolicited adverse events include lymphadenopathy and Bell’s palsy^12^.

Most side effects of the Oxford–AstraZeneca vaccine are mild, including injection site pain, fatigue, headache, muscle pain, feeling or being sick, fever, or chills 1 or 2 days after vaccination. Serious allergic reactions are rare^12^.

The Oxford–AstraZeneca vaccine was temporarily paused in several European countries due to reports of thromboembolic events in vaccinated individuals^13^. WHO Global Advisory Committee on Vaccine Safety has concluded that the available data do not suggest an overall increase in clotting conditions following the administration of the Oxford-AstraZeneca vaccine. As a result, the committee has recommended that the Oxford-AstraZeneca vaccine’s benefits outweigh its risks, with tremendous potential for preventing infections and deaths from SARS-CoV-2 infection^14^.

The use of digital technology to provide support, medical consultations, health-care services, and to track the spread of the infection without exposure to high-risk areas had a significant role in epidemic management^15^.

The Saudi Vision 2030 framework, released in 2017, has paved the path for digital transformation^16^. The use of such digital technologies including phone calls and electronic health records have played a vital role in supporting public health precautions and fighting SARS-CoV-2 transmission^17^.

Previous studies investigated the frequency of different adverse events of COVID-19 vaccines. However, the current study aims to compare these adverse events and their associated factors between the two main vaccines (Pfizer-BioNTech and Oxford-AstraZeneca vaccines) used in Saudi Arabia using 937 tele-consultation call center service.

## Objectives

1. To determine the frequency of immediate and short-term adverse events of the first and second dose of Pfizer-BioNTech and Oxford-AstraZeneca COVID-19 vaccines.
2. To compare the developed adverse events between the two studied vaccines and between the first and second dose of each vaccine.
3. To determine some risk factors associated with the development of these adverse events.

### Study design and setting

A cross-sectional study including all vaccination sites in Saudi Arabia was conducted in June 2021.

### Study participants

The study included individuals who completed 7 days after receiving the first or the second dose of either Pfizer-BioNTech or Oxford-AstraZeneca vaccines, 9–17 June 2021. The sample size was calculated using the formula: n = Z^2^ (pq)/d^2^, where p is the hypothesized prevalence of adverse events of COVID-19 vaccine and estimated at 20%, Z = 1.96, d = 5%. Accordingly, the calculated sample size was 245 (rounded to 250) for each dose of the vaccines. The Saudi COVID-19 database was used to collect the phone numbers of the included subjects. For good community representation of the sample, all the included Saudi regions were arranged, and then a systematic random sample technique was used to select the participants.

### Data collection

A structured and pre-coded questionnaire was used to collect data through mobile phones using 937 tele-consultation call center service. The medical consultation call center (937) is one of the Saudi Ministry of Health’ key transformation initiatives, aimed primarily at delivering timely and appropriate health services to every citizen. The center began operation early in 2017 and provides 24 h emergency and routine health care via telephone through a call-free number (937)^18^. The questionnaire was designed by the authors and validated using face validity. It included the following variables:

- Background variables, e.g., residence, age, sex, education, occupation.
- Type of vaccine received and the dose (first, second)
- Presence of immediate adverse events (within 15 min after receipt of the vaccine) including palpitation, loss of consciousness, dizziness, numbness, and nausea
- Presence of short-term local and systemic adverse events (within the first 7 days after receiving the vaccine), their time of onset, severity, and duration.
- History of chronic diseases, e.g., hypertension, obesity, diabetes, heart disease, kidney disease.

### Statistical analysis

Data analysis was performed using SPSS packages version 21. The frequency of both immediate and short-term adverse events for each dose and each vaccine were calculated. The short-term adverse events were categorized into local and systematic. The severity of adverse events was categorized into mild (does not interfere with daily activity), moderate (some interference with daily activity), severe (prevents daily activity), and emergency (usually requiring an emergency room visit or hospitalization)^19^. The difference between subgroups was tested using the Chi-squared test and reported using Odds ratios and confidence intervals. In addition, multivariate logistic analysis was done for the significant variables in the univariate analysis. P-value < 0.05 was considered to be statistically significant.

### Ethical consideration

The central institution review board of the Saudi Ministry of Health approved the study (IRB Log Number: 21-62M), therefore, all methods were performed in accordance with the relevant guidelines and regulations. Informed verbal consent was obtained from the participants before commencing the data collection. The data will not be used for purposes other than the study.

## Results

This cross-sectional study was conducted among 958 individuals 7 days after receiving Pfizer-BioNTech or Oxford-AstraZeneca vaccines during June 2021. Background variables of the studied vaccinated individuals are shown in Table 1. Gender distribution was almost equal for both vaccines and both doses. Most of those vaccinated with the first dose of Pfizer-BioNTech and Oxford-AstraZeneca were less than 55 years (93% and 85%, respectively). About one-third of the vaccinated people (33.7% and 30.4% of Pfizer-BioNTech and Oxford-AstraZeneca vaccines, respectively) had a secondary level of education, and more than half of them were unemployed (61.1% and 58.9% of both vaccines, respectively).

**Table 1:** Sociodemographic variables of the included vaccinated individuals.

Socio-demographic variables		Pfizer-BioNTech			Oxford-AstraZeneca		
		First dose (n = 230) N (%)	Second dose (n = 250) N (%)	Total (n = 480) N (%)	First dose (n = 228) N (%)	Second dose (n = 250) N (%)	Total (n = 478) N (%)
Gender	Male	124 (53.9)	125 (50.0)	250 (51.9)	103 (45.2)	125 (50.0)	228 (47.7)
	Female	106 (46.1)	125 (50.0)	230 (48.1)	125 (54.8)	125 (50.0)	250(52.7)
Age groups	< 55	215 (93.5)	41 (16.4)	256 (53.3)	196 (86.0)	77 (30.8)	273 (57.1)
	55+	15 (6.5)	209 (83.6)	224 (46.7)	32 (14.0)	173 (69.2)	205 (42.9)
Education	Illiterate	19 (8.3)	48 (19.2)	67 (14.0)	37 (16.2)	78 (31.2)	115 (24.1)
	Primary-Intermediate	50 (21.7)	56 (22.4)	106 (22.1)	44 (19.3)	51 (20.4)	95 (19.9)
	Secondary	86 (37.4)	75 (30.0)	162 (33.5)	91 (39.9)	54 (21.6)	145 (30.3)
	University graduate	71 (30.9)	65 (26.0)	136 (28.3)	53 (23.2)	58 (23.2)	111 (23.2)
	Post-graduate	4 (01.7)	6 (2.4)	10 (02.1)	3 (0.9)	9 (3.6)	11 (02.5)
Occupation	Unemployed	121 (52.6)	173 (69.2)	294 (61.3)	126 (55.3)	155 (62.0)	281 (58.8)
	Government employee	53 (23.0)	59 (23.6)	112 (23.3)	59 (25.9)	64 (25.6)	123 (25.7)
	Labour	53 (23.0)	10 (4.0)	63 (13.1)	40 (17.5)	29 (11.6)	69 (14.4)
	Business owner	3 (01.3)	8 (03.2)	11 (02.3)	3 (01.3)	2 (0.8)	5 (01.0)

Table 2 compares immediate and short-term adverse events of both studied vaccines. Immediate adverse events were reported among 1.04% and 2.09% for Pfizer-BioNTech and Oxford-AstraZeneca vaccines, respectively with no significant difference between both groups (X^2^ = 1.74, P = 0.19). Reported conditions included palpitation, dizziness, loss of consciousness, numbness, and nausea. All cases were treated within the vaccination sites and required no hospital admissions.

**Table 2:** Comparison of immediate and short-term adverse events of Pfizer-BioNTech and Oxford-AstraZeneca vaccines among studied participants.

Adverse events	Pfizer-BioNTech 1st and 2nd (n = 480)	Oxford-AstraZeneca 1st and 2nd (n = 478)	P-value	X^2^
	Frequency (%)	Frequency (%)		
Immediate	5 (1.04)	10 (2.09)	0.190	1.714
Systemic	197 (41.0%)	215 (45.0%)	0.218	1.515
Local	119 (24.8%)	47 (9.8%)	0.000	37.412
Fever	89 (18.5%)	130 (27.2%)	0.001	10.174
Fatigue	111 (23.1%)	120 (25.1%)	0.474	0.513
Headache	73 (15.2%)	82 (17.2%)	0.413	0.669
Vomiting	3 (0.5%)	8 (1.7%)	0.128	3.320
Diarrhea	6 (1.3%)	13 (2.7%)	0.103	2.661
Myalgia	99 (20.6%)	97 (20.3%)	0.899	0.16
Joint pain	39 (8.1%)	57 (11.9%)	0.050	3.835

Overall recipients of Pfizer-BionTech vaccine developed a higher percentage of local adverse events compared to recipients of AstraZeneca vaccine (24.8% versus 9.8%) (X^2^ = 37.41, P = 0.000). Whereas, no significant difference was recorded between both groups regarding the overall systemic adverse events (41.0% versus 45.0% respectively, X^2^ = 1.52, P = 0.218). However, AstraZeneca recipients developed higher percentage of fever (27.2%) compared to Pfizer recipients (18.5%) with a significant difference between both groups (X^2^ = 10.17, P = 0.001). Table 2 also shows that fatigue (23.1%) followed by myalgia (20.6%) represented the highest adverse events reported by Pfizer-BionTech vaccine. While among subjects vaccinated by AstraZeneca, fever followed by fatigue represented the highest reported adverse events (27.2% and 25.1% respectively).

Table 3 compares adverse events between the first dose of both studied vaccines. Concerning the first dose, recipients of AstraZeneca vaccine reported higher percentages for overall (P = 0.009) and all systemic adverse events (P < 0.05) compared to recipients of Pfizer-BioNTech vaccine with a significant difference between them (except for muscle ache P = 0.121). Whereas local adverse events were significantly higher among Pfizer-BioNTech recipients compared to AstraZeneca ones (23.5% versus 15.8% respectively, P = 0.03). Surprisingly, the reverse was observed for the second dose (Table 4).

**Table 3:** Comparison of local and systemic adverse events between first dose of both studied vaccines.

	Pfizer-BioNTech 1st dose (n = 231 )	Oxford-AstraZeneca 1st dose (n = 227)	X^2^	P-value
All systemic adverse events	107 (46.5)	134 (58.8)	6.892	0.009
Local adverse events	54 (23.5)	36 (15.8)	4.287	0.038
Fever	40 (17.4%)	99 (43.4%)	36.700	0.000
General fatigue	53 (23.0%)	84 (36.8%)	10.399	0.001
Muscle ache	52 (22.6%)	66 (28.9%)	2.405	0.121
Headache	36 (15.7%)	62 (27.2%)	9.067	0.003
Joint pain	14 (6.1%)	43 (18.9%)	17.142	0.000
Vomiting	1 (0.4%)	7 (3.1%)	4.634	0.031
Diarrhea	2 (0.9%)	9 (3.9%)	4.627	0.031

**Table 4:** Comparison of local and systemic adverse events between second dose of both studied vaccines.

	Pfizer-BioNTech 2nd dose (n = 250)	Oxford-AstraZeneca 2nd dose (n = 250)	X^2^	P-value
All systemic adverse events	90 (37.0)	81(32.4)	0.720	0.396
Local adverse events	65 (26.0)	11 (4.4)	45.246	0.000
Fever	49 (19.6%)	31 (12.4%)	4.821	0.028
General fatigue	58 (23.2%)	36 (14.4%)	6.341	0.012
Muscle ache	47 (18.8%)	31 (12.4%)	3.889	0.049
Headache	37 (14.8%)	20 (8.0%)	5.723	0.017
Joint pain	25 (10.0%)	14 (5.6%)	3.365	0.067
Vomiting	2 (0.8%)	1 (0.4%)	0.335	0.563
Diarrhea	4 (1.6%)	4 (1.6%)	0.000	1.000

Age group < 55 years, female gender, and obesity were significant risk factors for the development of systemic adverse events among the recipients of Pfizer-BioNTech vaccine (OR = 0.59, CI = 0.41–0.86, P = 0.005, OR = 1.69, CI = 1.17–2.44, P = 0.005, and OR = 1.85, CI = 1.16–2.95, P = 0.009 respectively). Other underlying comorbidities including hypertension, diabetes, Chronic Obstructed Pulmonary Diseases (COPD), cardiovascular diseases (CVD), and any chronic conditions were not significant risk factors for the development of these systemic adverse events (P > 0.05) Table 5.

**Table 5:** Risk factors associated with the development of systemic adverse events following Pfizer vaccine.

Variables		Absence of adverse events (n = 283)	Presence of adverse events (n = 197)	P value	OR (CI)
Age	55+	147 (65.6)	77 (34.4)	0.005	0.59 (0.41–0.86)
	< 55	136 (53.1)	120 (46.9)		
Gender	Male	162 (65.1)	87 (34.9)	0.005	1.69 (1.17–2.44)
	Female	121 (52.4)	110 (47.6)		
Presence of any chronic conditions		93 (57.1)	70 (42.9)	0.543	1.13 (0.77–1.65)
Obesity		41(46.6)	47 (53.4)	0.009	1.85 (1.16–2.95)
Hypertension		69 (60.5)	45 (39.5)	0.697	0.92 (0.60–1.41)
Diabetes		71 (65.1)	38 (34.9)	0.136	0.71 (0.46–1.11)
COPD		11 (47.8)	12 (52.2)	0.266	1.60 (0.69–3.71)
CVD		11 (55.0)	9 (45.0)	0.713	1.18 (0.48–2.91)

*COPD* chronic obstructed pulmonary diseases, *CVD* cardiovascular diseases.

Similarly, for recipients of Oxford-AstraZeneca vaccine, people aged < 55 years and females were significantly more likely to develop systemic adverse events by about 2.5 and 2 folds compared to those aged above 55 years and males (OR = 2.44 (1.67–3.55), P = 0.00 and OR = 1.82(1.27–2.63), P = 0.001), respectively. Other associated comorbidities were not significant risk factors for developing systemic adverse events among recipients of Oxford-AstraZeneca vaccine (P > 0.05) Table 6.

**Table 6:** Risk factors associated with the development of adverse events following AstraZeneca vaccine.

Variables		Absence of adverse events (n = 263)	Presence of adverse events (n = 215)	P-value	OR (CI)
Age	55+	138 (67.3%)	67 (32.7%)	0.000	2.44 (1.67–3.55)
	< 55	125 (45.8%)	148 (54.2%)		
Gender:	Male	143 (62.7%)	85 (37.3%)	0.001	1.82 (1.27–2.63)
	Female	120 (48.0%)	130 (52.0%)		
Presence of any chronic conditions		103 (58.5%)	73 (41.5)	0.240	0.8 (0.55–1.16)
Obesity		64 (55.2%)	52 (44.8%)	0.970	0.99 (0.65–1.51)
Hypertension		60 (58.3%)	43 (41.7%)	0.457	0.85 (0.54–1.32)
Diabetes		64 (60.4%)	42 (39.6%)	0.209	0.76 (0.49–1.17)
COPD		9 (50.0%)	9 (50.0%)	0.662	1.23 (0.48–3.16)
CVD		10 (52.6%)	9 (47.4%)	0.831	1.11 (0.44–2.77)

**Figure 1:**
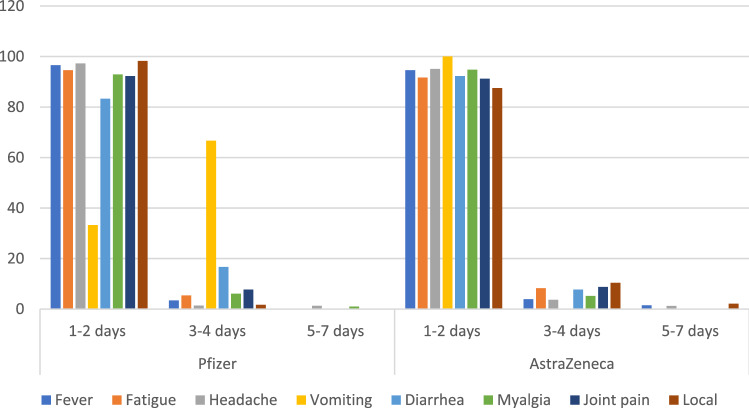
Onset of adverse events of studied vaccines among vaccinated subjects.

**Figure 2:**
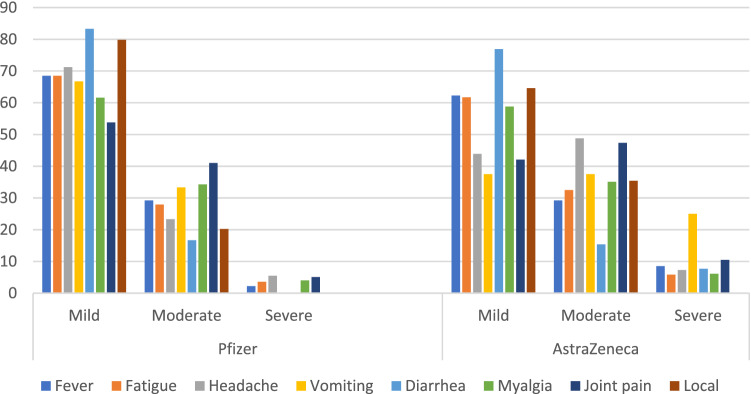
Severity of systemic and local adverse events of both studied vaccines among vaccinated subjects.

Figures 1 and 2 show the onset and severity of developed adverse events following administration of both studied vaccines. More than 96% of systemic adverse events developed within the first 2 days after vaccination. In addition, the majority of local and systemic adverse events of both vaccines were either mild or moderate in nature with no emergency cases.

## Discussion

This cross-sectional study was conducted in June 2021 to assess the frequency of immediate and short-term adverse events experienced by recipients of Pfizer-BioNTech and Oxford-AstraZeneca vaccines in Saudi Arabia.

The study revealed that immediate adverse events were reported among 1.04% and 2.09% for Pfizer-BioNTech and Oxford-AstraZeneca vaccines respectively with no serious events. Likewise, Chen et al. reported low rate of such immediate adverse events among 73,633 subjects from 14 articles included in a meta- analysis^20^.

The current study showed that recipients of Pfizer-BionTech vaccine developed a higher percentage of local adverse events compared to recipients of AstraZeneca vaccine with a significant difference between both groups (P = 0.000). This was in agreement with previous studies^21–23^. However, the frequency of these local adverse events was much lower than the rates reported in other studies (53.4% to 89.8%)^3,24–27^.

Concerning overall systemic adverse events, no significant difference was recorded between both studied vaccines (P = 0.218). These systemic adverse events following the administration of both studied vaccines respectively included mainly general fatigue (23.1% and 25.1%), fever (18.5% and 27.2%), myalgia (20.6% and 20.3%), and headache (15.2% and 17.2%).

These findings agree with those typically reported in randomized controlled trials and population-based studies in different countries, including Saudi Arabia^24,26–32^. However, the rates of these systemic adverse events vary in different studies. In our study, they ranged from 0.05% to 23.1% and 1.7% to 27.2%, following the administration of Pfizer-BioNTech and Oxford-AstraZeneca vaccines, respectively. This is in comparison to a range from 2 to 90% reported from Saudi Arabia^30^, 35–52% reported from Jordan^24^, 9.3–53.6% from Germany^3^, and 33.9–62.2% reported from the Czech Republic^32^. Our rates were also less than the rates reported in phase III clinical trials^3^.

On the other hand, our rates were higher than the results of a population-based study conducted in the United Kingdom, where only 25.4% of the study population had one or more systemic adverse events following the administration of Pfizer-BioNTech and Oxford-AstraZeneca vaccines^30^. However, another study from Saudi Arabia showed an overall rate of 50.3% of adverse events after any dose of Pfizer-BionTech^33^.

Comparing the first and second doses of both studied vaccine, recipients of the first dose of AstraZeneca vaccine reported higher percentages for overall systemic adverse events compared to recipients of Pfizer with a significant difference. Whereas the reverse was observed for the second dose. This was congruous with previous studies^21–23^. Conversely, Chen et al. found no significant differences on systemic adverse events between the first and second dose^20^.

Similarly, fever was reported among 17.3% and 43.2% of the recipients of the first dose of Pfizer and AstraZeneca vaccines respectively. This was observed among 19.6% and 12.4% for the second doses of both vaccines respectively. These rates were different from previous studies in Saudi Arabia, which reported fever among 18.5% and 4.3% for the first doses and 1.35 and 31.35 for the second doses of both vaccines respectively^25,28,31^.

The differences in the rates of the reported adverse events in different studies could be related to variation in the method of data collection, the background variable, different settings and the prevailing health conditions of the studied subjects^34,35^. The present study reported that age group < 55 years was significant risk factors for the development of systemic adverse events among the recipients of both studied vaccines.

This was consistent with the results of randomized control trials and population-based studies in Saudi Arabia and other countries^2,7,20,22,24,26,30,36,37^.

The increased probabilities of side effects among young adults could be explained by the potent generation of type I interferon (IFN-I) that initiates an effective immune response and is responsible for the occurrence of associated reactogenic adverse events^22,35^.

Also, confirming the results of other studies^22,28^, the rate of adverse events in our study was higher among females than males. The robust immune response and the lower pain threshold are suggested reasons to explain the gender-based differences in COVID-19 vaccine adverse events^22^.

Most of the adverse events reported in the present study developed within the first and second days following vaccination and were mild in nature. This was in harmony with other studies which reported that most of the adverse events tend to develop early and resolve within few days^2,7,22,26,27,30^. In addition, most studies reported that only a few cases suffered severe adverse events requiring medical attention^2,22,27^. Likewise, a systematic review of 11 published studies between Dec 2019 and 2020 indicates that COVID-19 vaccines can be safe and produce no serious adverse events^22^.

In conclusion, the most common reported systemic adverse events following the administration of both studied vaccines were general fatigue, fever, muscle pain, and fever. The majority of these adverse events were mild in nature and recede within few days. Additionally, they were more frequent in females and younger age group. Overall, systemic adverse events were more frequent following the first dose of AstraZeneca vaccine comparing to Pfizer vaccine, while the reverse was observed for the second dose.

The current study provided an insight on the adverse events following the administration of both COVID-19 vaccines approved in Saudi Arabia. The main limitations of the study included the cross-section design and self- reported adverse events. We tried to minimize these biases through contacting the participants within 7 days of vaccine administration to avoid recall bias and the nature of adverse effects were clearly explained to them. Other limitation of the study is the lack of information regarding past history of SARS-CoV-2 infection, which could be another risk factor for the development of adverse events. Additionally, the study stressed only on the short-term adverse events. Therefore, further cohort study is recommended to investigate the long-term adverse events following the administration of these COVID-19 vaccines.

## Data Availability

The data that support the findings of this study are available from the Saudi Ministry of Health but restrictions apply to the availability of these data, which were used under data sharing agreement policy for the current study, and are not publicly available. Data are, however, available from the corresponding author upon reasonable request and with permission from the central institution review board of the Saudi Ministry of Health.

## References

[1] European Centre for Disease Prevention and Control, Timeline of ECDC’s response to COVID-19. https://www.ecdc.europa.eu/en/covid-19/timeline-ecdc-response (2020) (Accessed 25 Feb 2021).

[2] World Health Organization, Covid-19 Vaccines. https://www.who.int/emergencies/diseases/novel-coronavirus-2019/covid-19-vaccines, (2021) (Accessed 20 Jan 2021).

[3] Polack FP, Thomas SJ, Kitchin N (2020). Safety and efficacy of the BNT162b2 mRNA Covid-19 vaccine. N. Engl. J. Med..

[4] World Health Organization, The Oxford/AstraZeneca COVID-19 vaccine: what you need to know. https://www.who.int/news-room/feature-stories/detail/the-oxford-astrazeneca-covid-19-vaccine-what-you-need-to-know (2021) (Accessed 12 Sept 2021).

[5] Food and Drug Administration, Development and licensure of vaccines to prevent COVID-19: guidance for industry. https://www.fda.gov/regulatory-information/searchfda-guidance-documents/development-and-licensurevaccines-prevent-covid-19 (2020) (Accessed 19 Jan 2021).

[6] Assiri A, Al-Tawfiq JA, Alkhalifa M, Al Duhailan H, Al Qahtani S, Dawas RA, El Seoudi AA, Alomran N, Omar OA, Alotaibi N, Almudarra SS (2021). Launching COVID-19 vaccination in Saudi Arabia: Lessons learned, and the way forward. Travel Med. Infect. Dis..

[7] Centers for Diseases Control and Prevention (CDC). Reactions and adverse events of the Pfizer-BioNTech COVID-19 vaccine (2021).

[8] Sahin U, Muik A, Vogler I, Derhovanssian E (2021). BNT162b2 vaccine induces neutralizing antibodies and poly-specific T cells in humans. Nature.

[9] World Health Organization, WHO Coronavirus Disease (COVID-19) Dashboard. https://covid19.who.int/ (2020) (Accessed 26 Sept 2021).

[10] Gianfredi V, Minerva M, Casu G (2021). Immediate adverse events following COVID-19 immunization. A cross-sectional study of 314,664 Italian subjects. Acta Bio Medica Atenei Parmensis.

[11] AARP, Health Conditions and Treatments, What Are the Side Effects of COVID-19 Vaccines?. https://www.aarp.org/health/conditions-treatments/info-2020/coronavirus-vaccine-side-effects/ (2020) (Accessed 17 Jan 2021).

[12] Centers for disease control and prevention, Local Reactions, Systemic Reactions, Adverse Events, and Serious Adverse Events: Pfizer-BioNTech COVID-19 Vaccine. https://www.cdc.gov/vaccines/covid-19/info-by-product/pfizer/reactogenicity.html, (2020) (Accessed 13 Dec 2020).

[13] NHS, Coronavirus (COVID-19) vaccines. https://www.nhs.uk/conditions/coronavirus-covid-19/coronavirus-vaccination/coronavirus-vaccine/ (Accessed 18 Sept 2021).

[14] Østergaard SD, Schmidt M, Horváth-Puhó E, Thomsen RW, Sørensen HT (2021). Thromboembolism and the Oxford–AstraZeneca COVID-19 vaccine: Side-effect or coincidence?. Lancet.

[15] Alghamdi NS, Alghamdi SM (2022). The role of digital technology in curbing COVID-19. Int. J. Environ. Res. Public Health..

[16] Hassounah M, Raheel H, Alhefzi M (2020). Digital response during the COVID-19 pandemic in Saudi Arabia. J. Med. Internet Res..

[17] Alghamdi, S. M., Alsulayyim, A. S., Alqahtani, J. S., Aldhahir, A. M. Digital health platforms in Saudi Arabia: determinants from the COVID-19 pandemic experience. In *Healthcare* Vol. 9, No. 11, 1517 (2021). 10.3390/healthcare9111517 PMC8618772 34828563

[18] Saudi Ministry of Health Portal. MOH Minister to Launch the 937-Service (MOH Emergency Call Center) Tomorrow. https://www.moh.gov.sa/en/937/Pages/default.aspx (2013).

[19] FDA, FDA Briefing Document. Pfizer-BioNTech COVID-19 vaccine. https://www.fda.gov/media/144245/download, (2020) (Accessed 17 Jan 2021).

[20] Chen M, Yuan Y, Zhou Y, Deng Z, Zhao J, Feng F, Sun C (2021). Safety of SARS-CoV-2 vaccines: A systematic review and meta-analysis of randomized controlled trials. Infect. Dis. Poverty.

[21] Food and Drug Administration, Pfizer-BioNTech COVID-19 Vaccine Emergency Use Authorization. https://www.fda.gov/emergency-preparedness-and-response/coronavirus-disease-2019-covid-19/pfizer-biontech-covid-19-vaccineexternalicon, (2021) (Accessed 12 Jan 2021).

[22] Klugar M, Riad A, Mekhemar M, Conrad J, Buchbender M, Howaldt HP, Attia S (2021). Side effects of mRNA-based and viral vector-based COVID-19 vaccines among German healthcare workers. Biology.

[23] Mathioudakis AG, Ghrew M, Ustianowski A, Ahmad S, Borrow R, Papavasileiou LP, Petrakis D, Bakerly ND (2021). Self-reported real-world safety and reactogenicity of covid-19 vaccines: A vaccine recipient survey. Life.

[24] Abu-Hammad O, Alduraidi H, Abu-Hammad S, Alnazzawi A, Babkair H, Abu-Hammad A, Dar-Odeh N (2021). Side effects reported by Jordanian healthcare workers who received COVID-19 vaccines. Vaccines.

[25] Al Bahrani S, Albarrak A, Alghamdi OA, Alghamdi MA, Hakami FH, Al Abaadi AK, Alkhrashi SA, Alghamdi MY, Almershad MM, Alenazi MM, El Gezery MH (2021). Safety and reactogenicity of the ChAdOx1 (AZD1222) COVID-19 vaccine in Saudi Arabia. Int. J. Infect. Dis..

[26] Alhazmi A, Alamer E, Daws D, Hakami M, Darraj M, Abdelwahab S, Maghfuri A, Algaissi A (2021). Evaluation of side effects associated with COVID-19 vaccines in Saudi Arabia. Vaccines.

[27] Hatmal MMM, Al-Hatamleh MA, Olaimat AN, Hatmal M, Alhaj-Qasem DM, Olaimat TM, Mohamud R (2021). Side effects and perceptions following COVID-19 vaccination in Jordan: A randomized, cross-sectional study implementing machine learning for predicting severity of side effects. Vaccines.

[28] El-Shitany NA, Harakeh S, Badr-Eldin SM, Bagher AM, Eid B, Almukadi H, Alghamdi BS, Alahmadi AA, Hassan NA, Sindi N, Alghamdi SA (2021). Minor to moderate side effects of Pfizer-BioNTech COVID-19 vaccine among Saudi residents: A retrospective cross-sectional study. Int. J. Gen. Med..

[29] Medicines & Healthcare Products Regulatory Agency. Coronavirus vaccine-weekly summary of Yellow Card reporting (Medicines & Healthcare Products Regulatory Agency, 2021).

[30] Menni C, Klaser K, May A, Polidori L, Capdevila J, Louca P, Sudre CH, Nguyen LH, Drew DA, Merino J, Hu C (2021). Vaccine side-effects and SARS-CoV-2 infection after vaccination in users of the COVID Symptom Study app in the UK: A prospective observational study. Lancet Infect. Dis..

[31] Mulligan MJ, Lyke KE, Kitchin N, Absalon J, Gurtman A, Lockhart S, Neuzil K, Raabe V, Bailey R, Swanson KA, Li P (2020). Phase I/II study of COVID-19 RNA vaccine BNT162b1 in adults. Nature.

[32] Riad A, Pokorná A, Attia S, Klugarová J, Koščík M, Klugar M (2021). Prevalence of COVID-19 vaccine side effects among healthcare workers in the Czech Republic. J. Clin. Med..

[33] Almohaya AM, Qari F, Zubaidi GA, Alnajim N, Moustafa K, Alshabi MM, Alsubaie FM, Almutairi I, Alwazna Q, Al-Tawfiq JA, Barry M (2021). Early solicited adverse events following the BNT162b2 mRNA vaccination, a population survey from Saudi Arabia. Prev. Med. Rep..

[34] Caubet JC, Ponvert C (2014). Vaccine allergy. Immunol. Allergy Clin. North Am..

[35] Hervé C, Laupèze B, Del Giudice G, Didierlaurent AM, Da Silva FT (2019). The how’s and what’s of vaccine reactogenicity. npj Vaccines.

[36] Ramasamy MN, Minassian AM, Ewer KJ, Flaxman AL, Folegatti PM, Owens DR, Voysey M, Aley PK, Angus B, Babbage G, Belij-Rammerstorfer S (2020). Safety and immunogenicity of ChAdOx1 nCoV-19 vaccine administered in a prime-boost regimen in young and old adults (COV002): A single-blind, randomised, controlled, phase 2/3 trial. Lancet.

[37] Voysey M, Clemens SA, Madhi SA, Weckx LY, Folegatti PM, Aley PK, Angus B, Baillie VL, Barnabas SL, Bhorat QE, Bibi S (2021). Safety and efficacy of the ChAdOx1 nCoV-19 vaccine (AZD1222) against SARS-CoV-2: An interim analysis of four randomised controlled trials in Brazil, South Africa, and the UK. Lancet.

